# Knowledge, Attitudes, and Practices Related to Ebola Virus Disease at the End of a National Epidemic — Guinea, August 2015

**DOI:** 10.15585/mmwr.mm6641a4

**Published:** 2017-10-20

**Authors:** Mohamed F. Jalloh, Susan J. Robinson, Jamaica Corker, Wenshu Li, Kathleen Irwin, Alpha M. Barry, Paulyne Ngalame Ntuba, Alpha A. Diallo, Mohammad B. Jalloh, James Nyuma, Musa Sellu, Amanda VanSteelandt, Megan Ramsden, LaRee Tracy, Pratima L. Raghunathan, John T. Redd, Lise Martel, Barbara Marston, Rebecca Bunnell

**Affiliations:** ^1^Division of Global Health Protection, Center for Global Health, CDC; ^2^National Center for HIV/AIDS, Viral Hepatitis, STD, and TB Prevention, CDC; ^3^International Ebola Taskforce, CDC; ^4^Division of Healthcare Quality Promotion, National Center for Emerging and Zoonotic Infectious Diseases, CDC; ^5^Sante Plus, Conakry, Guinea; ^6^Guinea Ministry of Health, Conakry, Guinea; ^7^FOCUS 1000, Freetown, Sierra Leone; ^8^Center for Drug Evaluation and Research, Food and Drug Administration.

Health communication and social mobilization efforts to improve the public’s knowledge, attitudes, and practices (KAP) regarding Ebola virus disease (Ebola) were important in controlling the 2014–2016 Ebola epidemic in Guinea (*1*), which resulted in 3,814 reported Ebola cases and 2,544 deaths.* Most Ebola cases in Guinea resulted from the washing and touching of persons and corpses infected with Ebola without adequate infection control precautions at home, at funerals, and in health facilities (*2*,*3*). As the 18-month epidemic waned in August 2015, Ebola KAP were assessed in a survey among residents of Guinea recruited through multistage cluster sampling procedures in the nation’s eight administrative regions (Boké, Conakry, Faranah, Kankan, Kindia, Labé, Mamou, and Nzérékoré). Nearly all participants (92%) were aware of Ebola prevention measures, but 27% believed that Ebola could be transmitted by ambient air, and 49% believed they could protect themselves from Ebola by avoiding mosquito bites. Of the participants, 95% reported taking actions to avoid getting Ebola, especially more frequent handwashing (93%). Nearly all participants (91%) indicated they would send relatives with suspected Ebola to Ebola treatment centers, and 89% said they would engage special Ebola burial teams to remove corpses with suspected Ebola from homes. Of the participants, 66% said they would prefer to observe an Ebola-affected corpse from a safe distance at burials rather than practice traditional funeral rites involving corpse contact. The findings were used to guide the ongoing epidemic response and recovery efforts, including health communication, social mobilization, and planning, to prevent and respond to future outbreaks or sporadic cases of Ebola.

Ebola-related KAP assessments were conducted in Sierra Leone (*4*), Liberia (*5*), Nigeria (*6*), and in one region in Guinea (*7*) during Ebola epidemics in 2014–2015. To learn more about Ebola-related KAP in Guinea as the nation’s epidemic waned following more than a year of Ebola education and prevention activities, several organizations conducted an Ebola KAP assessment across all administrative regions in August 2015. At that time, cumulative case counts varied substantially across the four natural regions of Guinea (Forest Guinea, Maritime Guinea, Middle Guinea, and Upper Guinea) (Figure); previously intense transmission had been controlled in the Forest Guinea region, but transmission persisted in the Maritime Guinea region (*8*). Various control measures were implemented, including case investigation and contact tracing, health communication about prevention practices, and specialized treatment units and burial teams to manage persons and corpses affected by Ebola.

**FIGURE:**
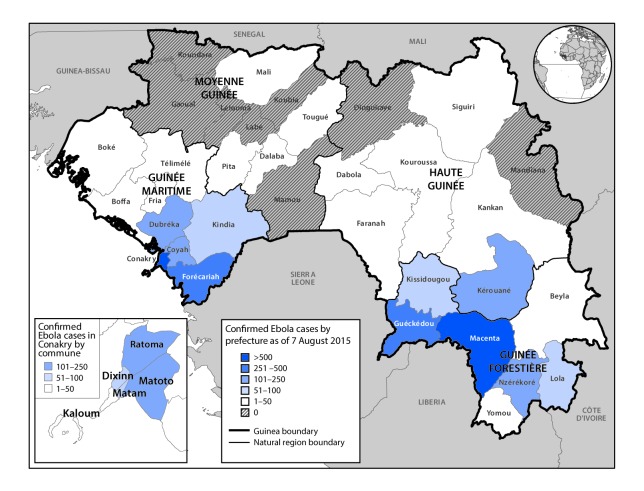
Cumulative confirmed cases of Ebola virus disease, by natural region* and administrative prefecture^†^ — Guinea, August 7, 2015 **Source:** Ebola situation reports by the World Health Organization. * Maritime Guinée = Maritime Guinea; Moyenne-Guinée = Middle Guinea; Haute-Guinée = Upper Guinea; Guinée Forestiere = Forest Guinea. ^^†^^ Of the sampled prefectures and urban communes, 12 reported 0–50 cumulative cases (Boffa, Boké, Dalaba, Dinguiraye, Fria, Kaloum, Kouroussa, Labé, Mamou, Tougué, Siguiri, and Yomou), and the rest reported 51 or more cumulative cases (Dixinn, Forécariah, Kindia, Kissidougou, Macenta, Matam, Matoto, Nzérékoré, and Ratoma). Four cases reported in Conakry prefecture could not be mapped to a commune.

The assessment employed a cross-sectional design using a multistage cluster sampling procedure. The 2014 Guinea Census List of Enumeration Areas served as the sampling frame for the random selection of 150 clusters across all eight administrative regions, which were grouped by the four natural regions of Guinea. Within each administrative region, prefectures were randomly sampled from among two strata defined by high (≥95) or low (<95) cumulative counts of confirmed cases that had been reported to the national Ebola surveillance system by May 2015. The sample was further stratified to include both urban and rural subprefectures. Districts within each subprefecture were randomly selected, and 20 households were selected from each cluster using a form of systematic random sampling known as the random walk method.^†^ In each selected household, two interviews were conducted; the first was with the head of household, and the second was with a randomly selected woman aged ≥25 years or a person of either sex aged 15–24 years. Interviews were conducted by locally trained data collectors using a free open-source set of tools to manage mobile data collection (https://opendatakit.org), installed on mobile devices. Data were analyzed using statistical software. For each record, weighted estimates adjusted for the probability of participant selection were calculated by applying a factor based on population size of the participant’s administrative region; 95% confidence intervals were generated for overall and regional data.

Data collection teams approached 6,699 persons, 6,273 (94%) of whom (from 3,137 households) consented to initiate the assessment. Among these, 5,733 (91%) persons who reported that they had heard of Ebola before the survey were asked questions for up to 60 minutes about Ebola through individual interviews that included closed- and open-ended questions in local languages, and rarely, in French. These respondents were considered to have completed the survey and were included in the analysis (Table 1). Overall, sociodemographic characteristics did not vary substantially by region, except that participants from Forest Guinea were more likely than other participants to report some formal education and Christian religious affiliations.

**TABLE 1:** Selected characteristics of respondents to a survey on Ebola virus disease knowledge, attitudes, and practices — Guinea, August 2015

Characteristic	Initiated survey (N = 6,273)* No. (%)	Completed survey (N = 5,733)^†^ No. (%)	% Completed survey, natural region			
			Maritime Guinea (n = 2,538)	Middle Guinea (n = 926)	Upper Guinea (n = 1,442)	Forest Guinea (n = 827)
**Administrative region**						
Conakry	920 (15)	915 (16)	36	—	—	—
Boké	664 (11)	581 (10)	23	—	—	—
Kindia	1,062 (17)	1,042 (18)	41	—	—	—
Mamou	400 (6)	366 (6)	—	40	—	—
Labé	579 (9)	560 (10)	—	60	—	—
Faranah	526 (8)	392 (7)	—	—	27	—
Kankan	1,142 (18)	1,050 (18)	—	—	73	—
Nzérékoré	980 (16)	827 (15)	—	—	—	100
**Sex**						
Male	3,164 (50)	2,937 (51)	52	44	53	54
Female	3,109 (50)	2,796 (49)	48	56	47	46
**Age group (yrs)**						
15–24	1,117 (18)	1,032 (18)	19	18	15	21
≥25	5,156 (82)	4,701 (82)	81	82	85	79
**Education**						
None	3,117 (53)	2,712 (50)	43	60	64	35
Some primary education	1,224 (21)	1,155 (21)	21	18	15	35
Some secondary education or higher	1,600 (26)	1,560 (29)	36	22	21	30
**Religion**						
Muslim	5,357 (86)	4,949 (87)	97	98	92	32
Christian	788 (13)	689 (12)	3	2	8	60
Other/None	93 (1)	68 (1)	0	0	0	8
**Occupation**						
Government/Office worker	364 (6)	358 (6)	8	5	5	4
Trader/Merchant	1,216 (20)	1,132 (20)	22	21	19	16
Farmer/Breeder	1,860 (30)	1,667 (29)	22	30	41	29
Police/Military/Guards	37 (1)	34 (1)	1	0	0	1
Student	629 (10)	600 (11)	12	12	6	12
Spiritual/Traditional healer	45 (1)	38 (1)	1	0	1	1
Skilled laborer	282 (5)	264 (5)	7	1	3	5
Other	1,230 (18)	1,120 (19)	18	23	17	25
Unemployed	554 (9)	478 (8)	9	8	8	7
**Heard of Ebola before interview**	**5,733 (93)**	**5,733 (100)**	**100**	**100**	**100**	**100**

* Denominator varied for those who initiated the survey with regard to education (N = 5,941), religion (N = 6,238), and occupation (N = 6,217).

^†^ Denominator varied for those who completed the survey with regard to education (N = 5,427), religion (N = 5,706), and occupation (N = 5,691).

Participants from the most heavily Ebola-affected regions (Forest Guinea and Martime Guinea) were more likely to have encountered Ebola response teams (61% and 72%, respectively), than were respondents from Middle Guinea (37%) and Upper Guinea (47%) (Table 2). Overall, 15% of participants perceived a high risk for acquiring Ebola; in Maritime Guinea, 25% of participants perceived a high risk. Most participants knew that Ebola is transmitted by contact with body fluids of infected persons (92%) or corpses (87%). However, the misconception that Ebola is transmitted by mosquito bites was reported by 49%, and this belief was reported by 66% of participants in Upper Guinea. Nearly all participants reported taking actions to avoid Ebola (95%), including more frequent handwashing (93%), avoiding contact with persons with suspected Ebola (44%), or avoiding crowds (22%).

**TABLE 2:** Knowledge, attitudes, and practices related to Ebola virus disease — Guinea, August 2015

Indicator	Response format	Overall*		Natural regions							
		No.	%	Maritime Guinea^†^		Middle Guinea^§^		Upper Guinea^¶^		Forest Guinea**	
				No.	% (95% CI)	No.	% (95% CI)	No.	% (95% CI)	No.	% (95% CI)
Encountered Ebola response teams in the past	Yes/No/DK	5,681	57	2,509	72 (69.8–73.3)	923	37 (33.6–39.9)	1,438	47 (44.1–49.3)	811	61 (57.5–64.3)
**Perceptions of personal risk for becoming infected with Ebola**											
No risk	Yes/No/DK	5,601	44	2,476	40 (38.4–42.3)	884	42 (39.2–45.8)	1,433	50 (47.6–52.8)	808	51 (47.4–54.4)
Low risk			27	2,476	23 (21.7–25.0)	884	30 (24.1–30.0)	1,433	28 (25.6–30.3)	808	35 (32.0–38.7)
High risk			15	2,476	25 (23.4–26.9)	884	9 (7.2–11.1)	1,433	8 (7.0–9.9)	808	5 (3.9–7.2)
Don’t know/Not sure			14	2,476	11 (10.1–12.6)	884	22 (19.1–24.6)	1,433	14 (11.8–15.5)	808	9 (6.8–10.7)
**Knowledge and perceptions about Ebola prevention and treatment**											
Preventable by avoiding contact with body fluids of infected persons	Yes/No/DK	5,715	92	2,526	91 (89.8–92.0)	925	94 (92.0–95.2)	1,440	94 (92.9–95.3)	824	89 (86.6–91.0)
Preventable by avoiding contact with corpse of persons who died from Ebola		5,708	87	2,524	86 (84.2–87.0)	922	93 (90.1–94.4)	1,440	87 (85.1–88.5)	822	83 (80.2–85.4)
Immediate treatment in health facility increases chance of survival		5,704	86	2,526	89 (87.6–90.0)	923	88 (85.5–89.7)	1,438	84 (82.0–85.8)	817	78 (75.4–81.0)
Immediate treatment in health facility reduces chance of Ebola spread		5,698	88	2,518	90 (88.4–90.8)	925	92 (89.7–93.3)	1,439	86 (84.4–88.0)	816	79 (76.1–81.7)
Male survivors should use condoms for at least 3 months to prevent sexual transmission^††^		5,237	46	2,396	44 (42.4–46.4)	746	39 (35.4–42.4)	1,341	49 (45.8–51.2)	754	57 (53.1–60.1)
**Misconceptions about Ebola transmission, prevention, and treatment**											
Transmissible by ambient air	Yes/No/DK	5,695	27	2,514	24 (22.6–26.0)	924	31 (27.6–33.6)	1,438	34 (31.5–36.3)	819	17 (14.1–19.1)
Can protect self from Ebola by avoiding mosquito bites		5,705	49	2,523	44 (42.3–46.1)	925	42 (39.0–45.4)	1,439	66 (63.8–68.6)	818	38 (35.1–41.7)
Preventable by bathing with salt and hot water		5,695	22	2,522	18 (16.6–19.6)	924	25 (22.1–27.7)	1,437	29 (26.6–31.2)	812	12 (9.5–13.9)
Can be successfully treated by spiritual or traditional healers		5,693	5	2,517	3 (2.7–4.1)	924	6 (4.6–7.8)	1,439	5 (3.9–6.1)	813	7 (5.1–8.5)
**Prevention practices used after learning about Ebola**											
Took some action to avoid Ebola infection	Yes/No/DK	5,537	95	2,452	97 (96.0–97.4)	900	93 (91.7–94.9)	1,407	92 (90.0–93.0)	778	95 (93.9–96.9)
Washed hands with soap and water more often	Open-ended, unprompted	5,240	93	2,370	94 (92.9–94.9)	840	91 (88.8–92.8)	1,288	94 (92.5–95.1)	742	95 (93.4–96.6)
Avoided all physical contact with those suspected of having Ebola		5,240	44	2,370	48 (46.1–50.1)	840	41 (37.4–44.0)	1,288	40 (36.8–42.2)	742	46 (42.2–49.4)
Avoided crowded places		5,240	22	2,370	24 (22.0–25.4)	840	16 (13.8–18.8)	1,288	27 (25.0–29.8)	742	13 (10.9–15.7)
**Intentions if family member suspected of having Ebola**											
Would send family member to an Ebola treatment center	Yes/No/DK	5,733	91	2,538	93 (92.1–94.1)	926	94 (92.2–95.4)	1,442	88 (86.2–89.6)	827	87 (84.6–89.2)
Would hide the family member from neighbors and health authorities		5,520	4	2,426	3 (2.5–3.9)	909	3 (2.1–4.5)	1,404	5 (3.6–5.8)	781	2 (1.3–3.5)
**Attitudes toward Ebola survivors^§§^**											
Survivors certified to be cured of Ebola could infect others through casual contact (e.g., hugging or shaking hands)	Yes/No/DK	4,637	17	2,093	13 (11.1–13.9)	768	25 (22.2–28.4)	1,135	21 (18.2–22.8)	641	12 (9.2–14.2)
Would not buy fresh vegetables from survivor certified by government to be cured of Ebola		5,417	28	2,367	21 (18.9–22.1)	903	40 (36.3–42.7)	1,372	36 (33.5–38.5)	775	16 (13.5–18.7)
Would not welcome survivor declared to be cured of Ebola back into community		5,468	19	2,402	14 (12.9–15.7)	911	26 (22.8–28.4)	1,365	28 (25.1–29.9)	790	6 (4.5–7.9)
Expressed one or more of the above attitudes toward Ebola survivors^¶¶^	Composite	5,029	44	2,203	35 (32.5–36.5)	871	58 (54.3–60.9)	1,283	55 (52.6–58.0)	672	30 (26.4–33.4)
Possible to survive and recover from Ebola	Yes/No/DK	5,703	72	2,523	81 (79.8–82.8)	925	74 (70.7–76.3)	1,437	58 (55.0–60.2)	818	69 (65.3–71.7)
Survivors could contribute to Ebola containment efforts		4,957	91	2,167	93 (92.2–94.4)	820	92 (90.5–94.1)	1,225	84 (81.9–86.1)	736	96 (94.8–97.6)
Survivors could educate community members about Ebola prevention	Open-ended, unprompted	4,516	62	2,022	58 (55.8–60.2)	757	60 (56.1–63.1)	1,029	63 (59.8–65.8)	708	71 (67.5–74.1)
Survivors could help care for persons suspected of having Ebola		4,516	37	2,022	46 (44.0–48.4)	757	35 (31.1–37.9)	1,029	39 (36.2–42.2)	708	18 (15.4–21.0)
**Intentions if family member died at home**											
Would wash or touch body if family member died	Yes/No/DK	5,460	8	2,416	5 (4.0–5.8)	870	11 (8.7–12.9)	1,403	8 (6.7–9.5)	771	10 (7.5–11.7)
Would wash or touch body if family member died of suspected Ebola		5,512	3	2,437	3 (2.7–4.1)	889	3 (2.0–4.2)	1,406	4 (2.5–4.5)	780	3 (2.0–4.6)
Would accept burial team if family member died of suspected Ebola		5,344	89	2,346	89 (88.0–90.6)	878	92 (90.6–94.2)	1,371	83 (81.0–85.0)	749	91 (88.8–93.0)
Would accept alternatives to traditional burials that do not involve physical contact with corpse if family member died of any cause		4,897	72	2,106	76 (74.4–78.0)	800	84 (81.4–86.4)	1,297	65 (61.9–67.1)	694	57 (53.4–60.8)
Observe burial from safe distance	Open-ended, unprompted	3,509	66	1,605	65 (62.8–67.4)	671	38 (34.3–41.7)	837	83 (80.5–85.5)	396	90 (87.5–93.3)
Have religious leader say a final prayer		3,509	54	1,605	67 (64.9–69.5)	671	54 (50.0–57.6)	837	34 (30.6–37.0)	396	58 (53.2–63.0)
Know the location of the burial site		3,509	22	1,605	21 (18.6-22.6)	671	11 (8.4–13.0)	837	18 (15.7–20.9)	396	66 (61.0–70.4)
Provide a name plate at the burial site		3,509	8	1,605	4 (3.0–5.0)	671	3 (1.6–4.0)	837	11 (8.5–12.7)	396	28 (23.1–31.9)
**Self-reported burial practices within past month of interview (for persons dying of any cause)**											
Participated in any burial ceremony in the past month:	Yes/No	5,532	20	2457	18 (16.0–19.0)	897	31 (27.5–33.5)	1,411	17 (14.8–18.8)	767	18 (15.6–21.0)
Washed the corpse	Open-ended, unprompted	1,082	6	431	1 (0.3–2.5)	274	3 (0.9–4.9)	237	5 (2.3–7.9)	140	16 (9.7–21.7)
Touched the corpse		1,082	4	431	4 (1.8–5.2)	274	5 (2.5–7.7)	237	5 (2.3–7.9)	140	19 (12.2–25.0)
Touched others at the burial ceremony (e.g., hug, handshake)		1,082	26	431	13 (9.4–15.6)	274	44 (38.3–50.1)	237	21 (15.5–25.9)	140	33 (25.1–40.7)
Cried over the corpse but did not touch it		1,082	27	431	17 (13.2–20.2)	274	30 (24.9–35.7)	237	42 (35.9–48.5)	140	22 (15.2–29.0)

**Abbreviations**: CI = confidence interval; DK = don’t know.

* Weighted percentages based on poststratification adjustments with probability proportional to population size of the participant’s administrative region.

^†^ As of August 2015, Maritime Guinea reported the total highest number of Ebola cases; all of its prefectures had reported cases, and it was the only natural region with active transmission (in Conakry and Forécariah prefectures) at the time of data collection.

^§^ As of August 2015, Middle Guinea was the region least affected by Ebola, and six of the 10 prefectures had never reported Ebola cases.

^¶^ As of August 2015, Upper Guinea had experienced low numbers of Ebola cases, and two of the eight prefectures had never reported Ebola cases.

** As of August 2015, Forest Guinea had no active transmission. However, it reported the first Ebola cases of the epidemic and eventually reported cases in all six prefectures.

^††^ Proportions of eligible participants who did not respond or replied “don’t know” were as high as 51.2% in Middle Guinea, 44.5% in Maritime Guinea, 41.4% in Guinea Upper, and 38.2% in Forest Guinea. These participants were not excluded from denominators when calculating percentages.

^§§^ Ebola survivors were defined as persons previously infected with Ebola who had been discharged from an Ebola Treatment Center and certified by government health officials to have been cured of the disease.

^¶¶^ Expressed one or more of the following attitudes about Ebola survivors: 1) survivors certified to be cured of Ebola could infect others through casual contact, 2) would not buy fresh vegetables from survivor certified by government to be cured of Ebola, and 3) would not welcome back into community a survivor declared to be cured of Ebola.

The majority of participants across all regions (91%) indicated they would send relatives with suspected Ebola to Ebola treatment centers. Most (72%) participants knew that one could survive and recover from Ebola, but such knowledge varied by region, and was lowest in Upper Guinea (58%) and highest in Maritime Guinea (81%). A minority of participants (17%) reported that survivors could infect others through casual contact such as hugging and shaking hands, that they would not buy fresh vegetables from shopkeepers who survived Ebola (28%), and that they would not welcome survivors into their communities (19%). Overall, 44% of participants expressed at least one of those three attitudes toward survivors, and these attitudes were more common in the less-affected regions (Middle Guinea [58%] and Upper Guinea [55%]) than in heavily affected regions (Maritime Guinea [35%] and Forest Guinea [30%]). In contrast, 91% of all participants expressed the opinion that Ebola survivors could contribute to Ebola control, such as through educating community members about Ebola prevention (62%) or caring for Ebola patients (37%) (Table 2).

When asked about intended burial preparations for family members suspected to have died from Ebola at home, only 3% of participants reported that they would wash or touch the body, and most stated that they would accept special Ebola burial teams (89%). Overall, 66% said they would prefer to observe corpses of family members who had died from Ebola from a safe distance at burials, but this attitude varied widely by region (Forest Guinea [90%]; Upper Guinea [83%]; Maritime Guinea [65%]; and Middle Guinea [38%]). Attitudes about other alternatives to touching Ebola-affected corpses also varied by region. When asked about intended burial preparations for family members who died of any cause at home, the majority of participants (72%) indicated they would accept alternatives that did not involve corpse contact, but this attitude was least common among respondents in Forest Guinea (57%). Among 1,082 (20%) participants who had recently attended burials of persons who had died from any cause, a minority reported washing (6%), touching (4%), or crying over the corpse without touching it (27%), but 26% reported touching other burial attendees. Participants from Forest Guinea were more likely to report recently washing (16%) or touching (19%) corpses than were participants from other regions (Table 2).

## Discussion

Eighteen months after the start of a devastating Ebola epidemic, most participants in this geographically diverse sample understood principal aspects of Ebola transmission and prevention, reported taking actions to reduce their risk for acquiring Ebola, and indicated they would use safer case management and burial practices for relatives with suspected Ebola. However, a substantial percentage of participants harbored misconceptions about Ebola transmission or expressed reticence about close proximity to Ebola survivors, including persons certified by the government to be cured of the disease. Although the World Health Organization declared Guinea to be Ebola-free by late 2015, clusters of Ebola cases occurred in 2016, partly through sexual transmission from survivors with persistence of Ebola virus in semen (*9*). These data underscore the value of ongoing health promotion efforts to prevent sporadic transmission or future outbreaks, including messaging that aims to reverse misconceptions about Ebola transmission and prevention, to clarify duration and modes of transmission from survivors, and to address stigma that survivors might face as they recover, rebuild their lives, and reintegrate into communities. Regional variations in the epidemic and related response activities might have resulted in the regional differences in attitudes and suggest that targeting health communication by region might be more effective than a uniform, national approach. Underlying differences in customs and traditions across different ethnic populations might have contributed to regional variation in attitudes and behaviors, especially regarding burials.

The assessment was the first national-level quantitative evaluation of Ebola-related burial practices among persons who attended a burial in West Africa during a period of ongoing Ebola transmission. It revealed that most participants would forsake traditional burial preparations involving washing or touching Ebola-affected corpses and would adopt safer practices without corpse contact. Compared with residents of other regions, residents of Forest Guinea were far more likely to indicate a preference for keeping a safe distance from Ebola-affected corpses. However, among the subset of persons who had recently attended burials for deaths from any cause, Forest Guinea residents were substantially more likely to have washed or touched corpses than were residents of other regions. The Forest Guinea region was the first region in the country to report Ebola cases and, unlike other regions, had contained its outbreak several months before the survey. This might explain why Forest participants reported a lower perceived risk for Ebola and might have reverted to traditional, high-contact burial practices for persons dying from causes other than Ebola. These findings underscore the observation that changes in cultural practices to combat highly infectious diseases such as Ebola might be transient, and that in-depth community engagement or new resources, such as cadres of professional body washers, might help prevent future transmission of infectious diseases related to corpse contact (*10*).

The findings in this report are subject to at least four limitations. First, because of the need to conduct the survey during the ongoing epidemic, interviewers did not validate the comprehension of some survey questions in French or other languages. Second, some participants might have provided socially desirable responses aligned to government recommendations rather than their actual opinions. For instance, government messages to encourage social distancing from Ebola-affected persons during the epidemic might have explained the reticence about close contact with Ebola survivors that some interviewers observed. Third, this analysis did not examine the relation between attitudes and exposure to health promotion interventions or messages. Finally, the sample was not nationally representative because of the partial randomization needed to intentionally oversample heavily affected areas, and the need to seek consent from heads of households, who were usually older men.

Despite their limitations, the mobile data collection tools permitted generation of preliminary findings that were shared with several organizations in Guinea within a few days of the interviews; this information was used to guide the ongoing response and health communication efforts, which contributed to eventual control of the epidemic. Such rapid KAP surveys, conducted during an outbreak, can provide important information for health communications efforts that can contribute to controlling an outbreak at its source, and thereby enhance global health security.

**Summary**

**What is already known about this topic?**

Assessments of knowledge, attitudes, and practices (KAP) in countries affected by the Ebola virus disease (Ebola) epidemic during 2014–2015 found that although most participants understood many aspects of Ebola transmission and prevention, misconceptions about the disease and transmission modes persisted. In Guinea, health officials suspected that traditional burial preparations and funeral rites involving corpse contact promoted transmission, but they lacked national-level data about these practices.

**What is added by this report?**

As the Ebola epidemic waned in Guinea, a KAP survey found that most participants understood Ebola causes, transmission, and prevention, but nearly half believed that Ebola could be transmitted by mosquitoes or ambient air. The majority of participants reported more frequent handwashing and avoiding physical contact with persons suspected of having Ebola. Nearly all participants reported they would seek specialized treatment for family members with suspected Ebola and would engage special burial teams if someone died from Ebola in their homes. More than half would observe Ebola-affected corpses from a safe distance that would avoid corpse contact, but there was considerable regional variation in that finding.

**What are the implications for public health practice?**

KAP information collected during an epidemic can yield data to guide response and recovery efforts, health education, and social mobilization. Future activities should aim to reverse misconceptions about Ebola transmission and prevention, clarify duration and modes of transmission from survivors, prevent stigmatization of Ebola survivors, and foster safer case management and burial practices.

## Footnotes

* https://www.cdc.gov/vhf/ebola/outbreaks/2014-west-africa/case-counts.html.

† A form of systematic random sampling that helps minimize survey administration cost and time by avoiding prior listing of all households in the enumeration area by beginning the process at a certain geographic point and following a specified path to select households to interview. https://unstats.un.org/unsd/demographic/meetings/egm/Sampling_1203/docs/no_2.pdf.
